# Advanced development and mechanism of sepsis-related acute respiratory distress syndrome

**DOI:** 10.3389/fmed.2022.1043859

**Published:** 2022-11-14

**Authors:** Huankai Gong, Yao Chen, Meiling Chen, Jiankang Li, Hong Zhang, Shijiao Yan, Chuanzhu Lv

**Affiliations:** ^1^Emergency and Trauma College, Hainan Medical University, Haikou, China; ^2^Key Laboratory of Emergency and Trauma of Ministry of Education, Hainan Medical University, Haikou, China; ^3^Department of Oncology, Southwest Hospital, Third Military Medical University (Army Medical University), Chongqing, China; ^4^Research Unit of Island Emergency Medicine, Chinese Academy of Medical Sciences (No. 2019RU013), Hainan Medical University, Haikou, China; ^5^School of Public Health, Hainan Medical University, Haikou, China; ^6^Emergency Medicine Center, Sichuan Provincial People's Hospital, University of Electronic Science and Technology of China, Chengdu, China

**Keywords:** sepsis, acute respiratory distress syndrome - ARDS, epithelial injury, endothelial injury, mechanism, biomarkers

## Abstract

The introduction of the Sepsis 3.0 guidelines in 2016 improved our understanding of sepsis diagnosis and therapy. Personalized treatment strategies and nursing methods for sepsis patients are recommended in the “Save Sepsis Campaign” in 2021. However, mortality in sepsis patients remains high. Patients with sepsis-related acute respiratory distress syndrome account for around 30% of them, with fatality rates ranging from 30 to 40%. Pathological specimens from individuals with sepsis-related ARDS frequently demonstrate widespread alveolar damage, and investigations have revealed that pulmonary epithelial and pulmonary endothelial injury is the underlying cause. As a result, the purpose of this work is to evaluate the mechanism and research progress of pulmonary epithelial and pulmonary endothelial damage in sepsis-related ARDS, which may provide new directions for future research, diagnosis, and therapy.

## Introduction

Sepsis, one of the most prevalent complications in the ICU, has a high fatality rate due to its complicated molecular underpinnings. Sepsis was described in 2016 as a “life-threatening organ failure produced by an unbalanced host response to infection” (1). Sepsis is frequently characterized by a dysregulated host response to invading pathogens; this systemic inflammatory response can result in disseminated intravascular coagulation, multiple organ dysfunction syndromes (MODS), and mortality (2). With the incremental development of sepsis diagnosis, treatment, and management over the last several decades, and the ongoing updating of recommendations, the mortality rate of sepsis has significantly declined (about 20–30 percent) (3). However, early detection of sepsis, prevention of multiple organ failure, and improved prognosis remain pressing concerns.

Sepsis frequently causes organ dysfunction and damage, such as acute kidney injury (AKI), acute lung injury (ALI), and ALI can be exacerbated by acute respiratory distress syndrome (ARDS). As a result, ARDS is often regarded as a deadly consequence of severe sepsis, with sepsis accounting for around 32% of all cases. The major histological hallmark of ARDS is severe diffuse alveolar damage, which is frequently driven by endothelial dysfunction and local inflammation. As a diverse illness, ARDS frequently manifests as sudden exacerbations of non-cardiogenic pulmonary edema, severe hypoxemia, and the requirement for mechanical ventilation (4, 5).

The focus of this review is on the pathophysiology and current research on sepsis-associated ARDS. It also goes through the biomarkers that play a role in sepsis-related ARDS, which may provide new directions for future research, diagnosis, and therapy.

## The potential mechanisms of sepsis-related ARDS

**Figure 1:**
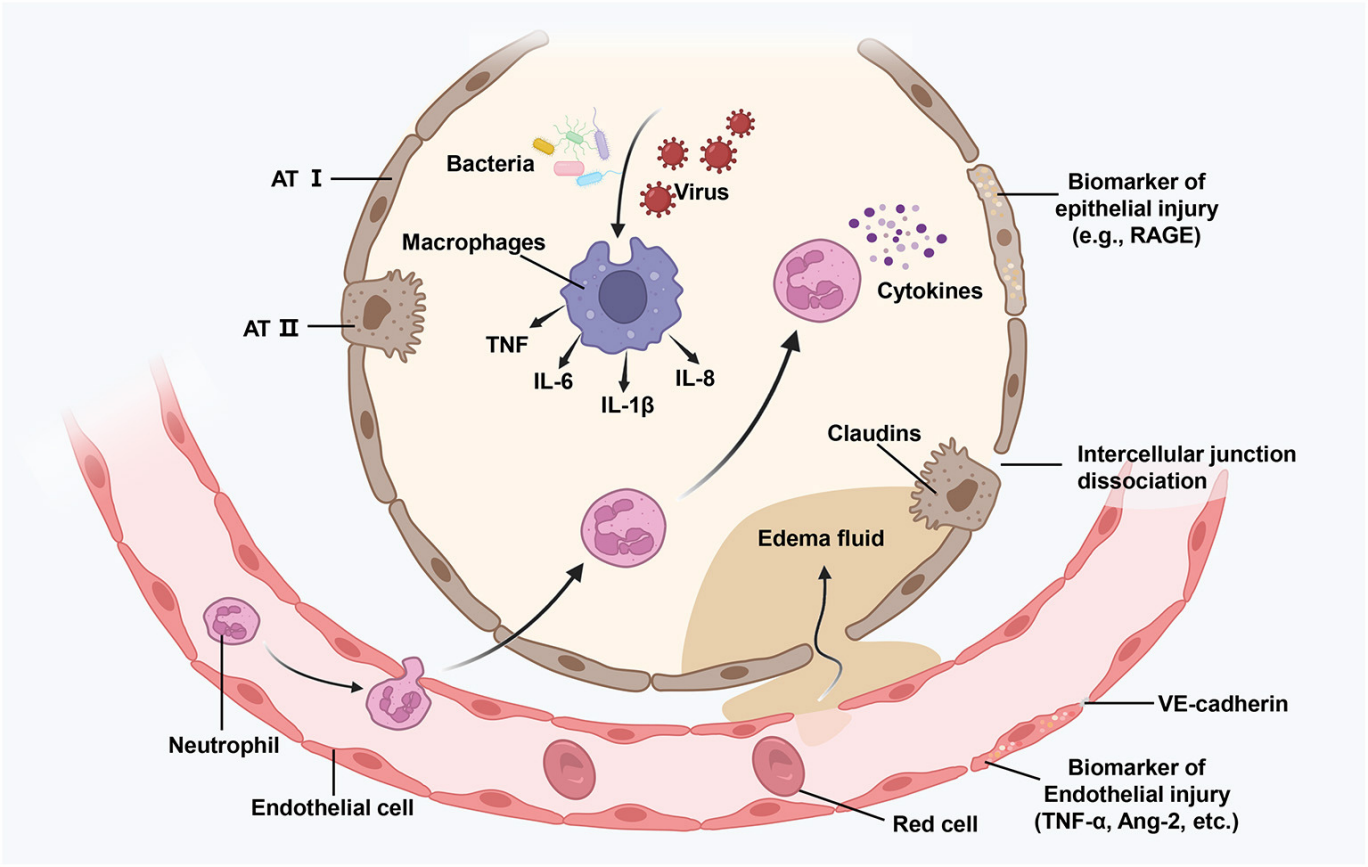
Illustration of an injured alveolus. Various damage factors (such as attack by bacteria and viruses) can directly or indirectly cause damage to the distal alveolar structure and the related microvascular regions. During the exudative phase, alveolar macrophages get activated, resulting in the release of powerful pro-inflammatory mediators and chemokines (such as TNF, IL-6, and IL-8) that promote the accumulation of neutrophils and monocytes. Activated neutrophils (such as cytokines) further promote damage by releasing toxic mediators. The resulting damage causes the loss of barrier functions as well as interstitial an intra-alveolar flooding. ATI, alvcolar type I cell; TNG, tumor necrosis factor; IL-6, interlcukin-6; Ang-2, angiopoietin 2; RAGE, receptor for advance glycation end-products.

A significant pathogenic characteristic of ARDS is the damage to vascular endothelial (VE) cells, alveolar epithelial cells, and epigenetics. However, the complex pathways underlying sepsis-related ARDS remain unknown. We have illustrated some potential mechanisms in Figure 1.

### VE injury

Although the nature and mechanism of endothelial injury in ARDS remain unknown, new research suggests that it is linked to inflammatory responses, VE-cadherin alteration, apoptosis, or other cell death pathways (such as pyroptosis and autophagy), and oxidative stress.

#### Inflammatory responses

In patients with sepsis, the immune system is usually in disequilibrium. Antigen-presenting cells activate a variety of signaling pathways between immune cells under external and internal stimulations, resulting in the release of inflammatory mediators such as interleukin (IL)-1, IL-6, IL-8, tumor necrosis factor-alpha (TNF-α), and the release of pro-inflammatory signals accelerates the vascular endothelial dysfunction, which, in turn, promotes the inflow of inflammatory cells (such as neutrophils, macrophages, monocytes, lymphocytes, and lymphocytes), forming a vicious pro-inflammatory cycle, which ultimately aggravates and amplifies lung or systemic inflammation (6, 7). Therefore, pathogen-derived inflammatory mediators and activated immune cells not only trigger immunological responses but also cause host cell harm in sepsis.

The activation and destruction of endothelial cells can result in the production of pro-inflammatory signaling molecules (such as platelet-activating factor, angiopoietin 2, tumor necrosis factor, VE growth factor, inflammasome product IL-1, and others) and the accumulation of leukocytes, resulting in the recruitment of neutrophils and macrophages, activation of alveolar epithelial cells, and effector T cells (5, 8), Leukocyte aggregation is most commonly seen in the form of neutrophil-platelet aggregates, which have complicated thrombo-inflammatory properties (9). This can lead to increased protein permeability in the pulmonary vascular system, which can lead to hypovolemia and multiple organ failure. Neutrophil extracellular traps (NETs), for example, have been linked to the disruption of alveolar-capillary and epithelial barriers in recent research on acute lung damage and ARDS, as well as having inflammatory effects on the lung and other organs (10).

In addition, alveolar macrophages (AM) can cooperate with other immune cells to regulate lung inflammation, and AM cell death plays an important role in the development process of lung inflammation (11–14). On one hand, a variety of proinflammatory cytokines (e.g., inflammasomes and IL-1 β) can activate or amplify the lung injury response of macrophages, T cells, and other immune cells (15–17). On the other hand, AM cell death or pyroptosis promotes neutrophil migration into the lungs, increases the concentration of cytokines (e.g., IL-6, TNF-α, and IL-1β) in the alveoli, and aggravates lung injury (18). Thus, the interaction between inflammation and cell death is expected to further affect and accelerate the progression of ARDS.

In addition, the bacterial endotoxin lipopolysaccharide (LPS), which is a typical activator of sepsis-induced lung injury, can activate the overexpression and release of a variety of pro-inflammatory proteins, resulting in severe cellular or organ damage.

#### Disruption of VE-cadherin

The synergistic effect of VE-cadherin and endothelial receptor kinase (TIE2), which is regulated by VE protein tyrosine phosphatase, ensures the integrity of VE cells (VE-PTP) (4, 19, 20). Several variables influence and regulate the activity of VE-cadherin and the stability of adhesive junctions, including cytoskeletal interactions, GTPases, phosphorylation, and dephosphorylation (21). Dissociation of VE-PTP from VE-cadherin has been linked to enhanced alveolar-capillary permeability in inflammatory acute lung damage, as well as endothelial cell junction relaxation and inflammatory alveolar protein leakage, according to research (9, 22). The inflammatory response can destabilize VE-cadherin by releasing a range of small molecules (such as angiopoietin 2 and VE growth factor). Furthermore, actin filaments that generate tension and actin stress fibers that generate tension work together to influence the stability of VE cell connections (23, 24). Loss of intercellular adhesion during actomyosin contraction causes gaps to emerge between endothelial cells. These mechanisms combine to promote endothelial and epithelial permeability, which contributes to edematous fluid buildup and hypoxemia.

#### Apoptosis or other cell death pathways

The clinical significance of neutrophil apoptosis sensitivity in the etiology of ARDS is unknown. ARDS has been linked to the influx of neutrophils into the alveoli in several studies. Apoptotic neutrophils amass in the alveoli as a result of decreased AM proliferation, resulting in secondary necrosis and the release of inflammatory mediators (25, 26). In addition, the buildup of a significant number of neutrophils plays a key role in the release of additional inflammatory mediators and pro-inflammatory factors throughout the ALI process (27).

Necroptosis has been studied extensively as a crucial contributor to apoptosis in ARDS. Under the stimulation of a death signal, receptor-interacting protein kinase 1 (RIPK1) and RIPK3 govern necrotic cell death, which can be suppressed by necrostatin-1 (NEC-1) (28, 29). The presence of HMGB1 in bronchoalveolar lavage of patients with acute lung damage generally indicates necroptosis. Similarly, numerous intracellular bacteria and viruses elicit necroptosis in the lungs and have a role in sepsis-induced ARDS development (30).

Pyroptosis plays a role in the pathophysiology of ARDS as well. Lipopolysaccharide, which is primarily mediated by the cysteine protease (Caspase) family, affects endothelial cell pyroptosis in animal models (such as Caspase-1, Caspase-4, Caspase-11). Gasdermin D can be cleaved by activated Caspase-1 to generate the N-terminus or C-terminus of Gasdermin D, which is a direct executive protein of pyroptosis. Gasdermin D's N-terminus attaches to phospholipid proteins on the cell membrane, creating a hole that allows a flood of inflammatory substances to escape the cell (31).

In conclusion, apoptosis overexpression is important in the development of acute lung damage and ARDS.

#### Oxidative stress

Oxidative stress is frequently associated with ARDS. A large number of cytokines and inflammatory cells can be released during the inflammatory response of sepsis, and a large number of reactive oxygen species (ROS) can be generated through the oxidative stress response, causing varying degrees of damage to the structure and function of cells, such as mitochondrial damage (32). As per a past report, when cells are exposed to bacteria, leukocyte respiration increases, which kills the pathogens by producing ROS, superoxide, hydrogen peroxide, and hydroxyl radicals (33). NADPH oxidase (NOX) is an enzyme that uses NADPH to catalyze the reduction of oxygen to produce superoxide (34). NOX is commonly referred to as a “professional ROS producer”. Currently, seven NOX isoforms are known, namely, NOX1, NOX2, NOX3, NOX4, NOX5, Duox1, and Duox2 (35). Among these, only NOX1, NOX2, and NOX4 are expressed in the vasculature, and all of them have been implicated in ROS-mediated vascular diseases (36). The ROS produced by LPS exposure has been demonstrated to be NOX1-dependent in macrophages and NOX2-dependent in LPS- challenged lungs (37, 38). The LPS activating the toll-like receptor 4 (TLR4) receptor induces NOX-mediated ROS generation (39), which in turn activates pro-inflammatory signaling factors such as the TNF-α and NF-κB (40, 41). Protein interaction with C-kinase 1 (PICK1) affects pulmonary vascular glutathione synthesis by influencing the substrate-specific component xCT of the pulmonary cystine/glutamate transporter, resulting in severe oxidative stress, according to an animal study on sepsis (42).

### Alveolar epithelial injury

One of the key hallmarks of ARDS is alveolar epithelial injury, and the severity of epithelial cell injury is a significant factor in ARDS severity.

The early stage of lung injury, commonly known as the exudative phase of ARDS, is characterized by innate immune cell-mediated disruption of the alveolar endothelial cell barrier and accumulation of protein-rich edema fluid in the alveolar interstitium and alveolus (5). Macrophages in the alveoli generate pro-inflammatory substances, which attract neutrophils, monocytes, and macrophages, as well as activate alveolar epithelial cells and effector T cells, causing inflammation and tissue damage (43). Second, alveolar endothelial cells with enhanced permeability allow proteins and fluids to collect in the pulmonary interstitium, resulting in interstitial edema. The edema fluid is transmitted to the alveolar fluid at this moment due to the alveolar epithelium's normal tight barrier being compromised (4).

#### Dissociation of intercellular junctions

When endothelial cells mount a proinflammatory or procoagulant response to infection in neighboring epithelial cells, the alveolar epithelial-endothelial barrier occurs independently of endothelial cells, according to Kirsty et al. (44). Barrier injury, on the other hand, is linked to the breakdown of epithelial cells' tight junctions. The alveolar epithelium's tight junctions are critical for regulating fluid in the lung's distal space, and transmembrane tight junction proteins called Claudins play a significant role (45).

Claudins 3, 4, 5, 7, 8, 15, and 18 were all expressed in the distal lung. Claudins 3, 4, and 7 are mostly found in alveolar type II cells, whereas claudin-5 is found in nearly all alveolar epithelial cells (46). The loss of the tight junction protein claudin-4 is one of them, and it's linked to barrier destruction (44). Claudin-5 disrupts the function of the alveolar epithelial barrier by interfering with the interaction of claudin-18 with the scaffold protein ZO-1 (45).

#### Epithelial cell death

Lung epithelial cells are usually regarded as the lung's first line of defense, and epithelial cell death is the most prominent aspect of alveolar damage in ARDS, which can be induced directly by bacterial and viral invasion, acidic media, hyperoxia, hypoxia, and mechanical alterations (47, 48). Inflammatory macrophages can promote cell death through methods such as the production of tumor necrosis factors and similar apoptosis-inducing ligands, while neutrophil-derived mediators can induce cell death through many pathways, including the release of TNF (49, 50).

### Epigenetics

The term “epigenetics” describes the regulatory systems that manage gene expression but are unrelated to changes in the DNA sequence. These changes include non-coding RNA control of transcription, DNA methylation, and histone changes (51). The confluence of genetics and environment is where epigenetic alterations, which affect gene expression in response to external stress, occur. Epigenetic control may be the key factor in the pathogenesis of sepsis, as per several recent types of research on immunology and human sepsis (52).

#### Epigenetics and sepsis-related immune suppression

Anti-inflammatory and pro-inflammatory symptoms are present in the early stages of sepsis, but, as the condition progresses, immunosuppression frequently predominates, which increases the risk of subsequent infection and death. Immunosuppression induced by sepsis is a complicated phenomenon. Endotoxin tolerance or the body's inability to respond to bacterial endotoxin is one of the current markers of sepsis-associated immunosuppression. Numerous *in vitro* studies support the idea that epigenetic changes are essential for the development of endotoxin tolerance. In researches by El Gazzar et al. (53, 54), it was demonstrated that the TNF promoter was methylated in monocytes in the stationary phase. The TNF promoter is quickly demethylated upon initial endotoxin exposure, inducing an immunological response. However, the TNF promoter is bound by the histone methyltransferase G9a throughout the endotoxin-tolerance phase, resulting in recurrent methylation of the TNF promoter, which eventually renders the TNF promoter insensitive to endotoxin activation. miRNAs may also play a role in endotoxin tolerance, where miR-125b, miR-146a, miR-221, and miR-579 are involved in controlling the transcriptional expression of TNF (55, 56).

#### Epigenetics and ARDS

ARDS is a common complication of sepsis, and past studies have demonstrated that DNA methylation plays an important role in its pathogenesis. In an LPS-induced ARDS rat model, the level of 5-methylocytosine was increased, which confirmed the increased DNA methylation level (57). In an epigenomic analysis of lung tissues, more than 1,700 genes exhibited methylation differences (58). Of the 42 differential methylation genes associated with MAPK signaling, seven were found to be associated with ARDS (59). In their recent study, Chen et al. (60) confirmed the METTL3-mediated abnormal n6-methyladenosine mRNA expression in the septic lungs. Moreover, decreased METTL3 levels could exacerbate lung endothelial injury and inflammatory responses in sepsis-related ARDS. These findings provide a new direction in the research of whether sepsis-related ARDS can develop METTL3 as a biomarker or as a therapeutic intervention point.

## Biomarkers of sepsis-related ARDS

A variety of biomarkers can be used to determine the severity of ARDS and the characteristics of each stage. The ideal biomarker would be based on the more precise pathophysiological pathways that have been investigated thus far. It must be extremely dependable, repeatable, disease-specific, and sensitive. The procedure is easy and low-cost in clinical practice, and short-term volatility must also be considered. Blood or plasma, urine, feces, bronchoalveolar lavage fluid, cerebrospinal fluid, bone marrow, and other clinical test specimens are currently used. It can reflect the damage or activation of epithelial cells, endothelial cells, or the coagulation system in the ARDS inflammatory response by detecting the change of a single biomarker in the specimen, which can aid in the diagnosis of the disease or the judgment of the curative effect in the treatment of the disease. Predict the present patient's cure rate or fatality rate.

### Interleukin-6

Interleukin-6 (IL-6) was first discovered in 1986 under the name B cell-stimulating factor. T cells produce it, but it can also cause B cells to create antibodies. IL-6 has become a key inflammatory regulator since its discovery, and it is secreted by a variety of cells, the majority of which are found in inflammatory, infectious, and neoplastic disorders (61). IL-6 levels have been discovered to be elevated in critical conditions including sepsis and ARDS, and studies have demonstrated that it plays a key role in the disease's progression. Because IL-6 is required for clearing infections in the immune process and plays an active role as an anti-inflammatory or protective factor in most cases, future research can use IL-6 concentrations in the blood or lung as a biomarker to explain the current status of the disease.

Classical signaling and trans-signaling are the two basic types of IL-6 signaling. In recent years, a third transduction mechanism known as trans-presentation has been found. IL-6 forms the IL-6-IL-6R complex with membrane-bound IL-6R, which subsequently binds to gp130 to form signal transduction via the JAK-STAT pathway in traditional signaling. Only a few types of cells, most notably hepatocytes and some leukocytes such as macrophages and T-cell subsets, express IL-6R, but gp130 is expressed by all cell types. IL-6 does not bind to gp130 on its own; it must first form a complex with IL-6R. This signal transduction pathway is primarily responsible for IL-6's anti-inflammatory and antibacterial actions (62). IL-6R is cleaved off the cell surface and alternatively spliced to produce the soluble receptor sIL-6R in trans-signaling. The capacity of sIL-6R to bind to IL-6, and the resulting IL-6-sIL-6R complex to bind to gp130, can be performed on cells without IL-6R, extending the range of IL-6 on target cells and explaining IL-6's versatility. Protease disintegrin and metalloproteinase domain-containing protein 17 (ADAM-17), which is activated during inflammation or infection, is the major enzyme capable of completing this cleavage event, hence the pro-inflammatory effect is mostly mediated through trans-signaling (63). Trans-presentation is the third signal transduction mode. IL-6 is expected to be delivered to the plasma membrane after engaging with antigen-specific dendritic cells (DCs) and binding to IL-6R on DCs. Under the joint action of transforming growth factor-beta 2(TGF-2) and the IL-6-IL-6R complex on the surface of dendritic cells, it binds to gp130 on the surface of T cells and activates pathogenic Th17 cells (64).

In the course of sepsis or ARDS, IL-6 plays a significant role, and its management can have a favorable influence on the condition. The current study focuses on blocking IL-6, and IL-6R, neutralizing gp130, and interfering with JAK-STAT signaling, and it has yielded some promising results (62). The research on IL-6 as a biomarker in critical diseases, particularly ARDS and COVID-19, has made some headway. It is difficult to determine the concentration of circulating IL-6 and interpret the results. The cytokine peaks at different times in different disorders, making the sample time more restrictive. Changes in circadian rhythm, exercise, certain medicines, and immunometabolism comorbidities can all impact IL-6 levels and release in the bloodstream (65). There are further needs for sample processing, as il-6 and other cytokines are produced from blood cells over time, altering results (66). COVID-19 has swept the globe in the last 2 years, and its severe sufferers are likely to develop ARDS. Due to the lack of a common definition, some of the data gathered in the current COVID-19 research on IL-6 may have varied results. Part of the explanation for this disparity could be the use of clinically-based IL-6 tests, which are notoriously less sensitive than currently available research-grade assays. A recent series of cytokine-focused prospective studies in critically ill COVID-19 patients found that IL-6 concentrations were significantly higher than previously reported using clinical IL-6 measurements, both in absolute terms and relative to other inflammatory airway conditions like ARDS (67–69). IL-6 was found to act as an independent predictor of 28-day mortality in sepsis patients, showing superior predictive power to procalcitonin and hypersensitive C-responsive proteins as per meta-analysis. With the combined application of IL-6 and neutrophil-lymphocyte ratio as a predictive model, the predictive power of death risk in sepsis patients was significantly improved (70). This aspect benefits clinicians for more appropriate and accurate management of patients with sepsis.

### Angiopoietin 2

Davis et al. (71) found the vascular receptor tyrosine kinase Tie-2 and its ligand angiopoietin 1 (Ang-1) in the mid-1990s and then used homology screening of a cDNA library to find angiopoietin 2 (Ang-2). Tie-2 receptors can bind to both Ang-1 and Ang-2. Tie-2 receptors are activated and phosphorylated after Ang-1 binds, promoting blood vessel integrity and growth. Ang-2 functions as an antagonist of Ang-1, binding to Tie-2 receptors competitively and blocking Ang-1's actions, boosting inflammatory responses and capillary leakage. Because Ang-2 is implicated in the pathophysiology of a variety of disorders, it could be used as a therapeutic target, and certain Ang-2-targeted therapies have been demonstrated to be effective (72, 73).

ARDS is a leading cause of morbidity and mortality in patients around the world, and despite effective antibiotic treatment, pathogen-body interactions can lead to increased pulmonary endothelial cell permeability, which can lead to protein exudation and edema, and eventually life-threatening lung failure. Ang-2 is the principal cause of enhanced permeability in lung endothelial cells. Ang-2 has been validated as a biomarker for sepsis and ARDS risk assessment in several prior studies (74, 75). Despite considerable knowledge of Ang-2's expression and activity in pulmonary circulation, its significance in the development of pneumonia and ARDS is unknown. Gutbier et al. (76) confirmed that Ang-2 levels were significantly greater in ARDS patients than in healthy people in a prospective analysis of two different cohorts and that using Ang-2 as a particular biomarker could improve the CURB-65/CRB-65 grading system accuracy. International recommendations indicate the CURB-65 and CRB-65 scores as predictors of pneumonia fatality (77). It was discovered that Ang-1, Ang-2, and its receptor Tie-2 were considerably expressed in the lung tissue of patients with pneumonia after evaluating the lung tissue of the deceased patient. The Ang-1 protein is found in a variety of types, including lung parenchymal cells, endothelial cells, and epithelial cells. Ang-2 and Tie-2 proteins, on the other hand, were only found in pulmonary VE cells (76). Villar et al. (78) discovered that Ang-2 plays an essential role in ARDS prediction in septic patients in a multicenter observational study in Spanish intensive care units. In a prospective study, Ang-2 demonstrated a large independent association between severe sepsis and organ injury (79). These studies implied that Ang-2—a biomarker of endothelial dysfunction and damage—may play an important role in future studies on predicting the treatment and prognosis of sepsis.

### Receptor for advanced glycation end products

RAGE (receptor for advanced glycation end-products) is an immunoglobulin superfamily multi-ligand pattern recognition receptor. It is mostly found in membrane-bound and soluble forms (sRAGE). Membrane-bound forms can identify a wide range of receptors, activate transcription factors *via* binding to receptors, and enhance pro-inflammatory factor production. The soluble form is a decoy receptor that suppresses membrane RAGE activation competitively (80). RAGE is extensively expressed in lung tissue under normal circumstances (81). By evaluating the level of RAGE in bronchoalveolar lavage fluid and serum of rats and people with acute lung damage, Uchida et al. (82) confirmed that RAGE is a biomarker of type I alveolar epithelial cell injury and a significant inflammatory mediator as early as 2006. This is critical because epithelial cell injury and inflammatory responses are both involved in the ARDS process, and RAGE is involved in both of these routes (80). Following research, it was discovered that sRAGE is linked to the severity of ARDS (83–85).

Two subtypes of sARGE, known as cRAGE and esRAGE, have been separated in recent years (for endogenous secretory RAGE). To facilitate the shedding of sARGE, inflammatory factors are amplified and result in the formation of cRAGE, which is created on the surface of the cell membrane by proteolytic cleavage at the extracellular and transmembrane boundaries (86). Less than 25% of the total circulating sRAGE is created by alternate splicing of the RAGE pre-mRNA (87, 88), and the specific process governing esRAGE production is currently unknown. Studies have shown that increased sRAGE levels during acute illness predict 90-day death in ARDS patients (89). RAGE is thought to be substantially overexpressed in the lung epithelium and that RAGE signaling may play a key role in the clinical symptoms of lung injury (90). High levels of sRAGE are also linked to potential mortality in sepsis (91). Theoretically, respiratory virus illnesses like Covid-19, which are currently wreaking havoc worldwide, may also be connected. The next study objective may be to construct risk classification and sRAGE thresholds for sRAGE levels in clinical practice, which will help sRAGE as a biomarker to better serve the clinic.

In summary, past studies on the biomarkers of sepsis-related ARDS were mostly focused on two aspects: the biomarkers of VE injury and alveolar epithelial injury. The common ones included IL-6, Ang-2, and sRAGE. Despite the lack of any substantial evidence supporting the specificity of these biomarkers for such diseases, recent studies have hinted that the levels of these biomarkers are associated with an increased risk of sepsis-related ARDS development (92–94). In recent years, the researchers turn their attention to the genetic basis of these relationships. Recent studies suggest that genomic or transcriptome-based biomarkers may facilitate the establishment of predictive or prognostic stratification approaches for sepsis-associated ARDS and may, thereby, facilitate the development of novel therapeutic targets. For example, epigenetic variants and circulating microRNA have become potential biomarkers for the diagnosis or prognosis of sepsis-associated ARDS (95). However, they are possibly limited by various factors such as the sample size, ethnicity, and phenotypic heterogeneity. The current study did not detected any exact association of these novel biomarkers with sepsis-related ARDS (96). Nevertheless, this finding also provides a new direction for further research.

## Conclusions

Sepsis-related ARDS is an inherently heterogeneous clinical syndrome. Several potential biomarkers have been investigated so far, with no single biomarker yet identified that can specifically reliably diagnose this disease. Current research indicates that biomarker combinations that respond to different aspects (such as epithelial and endothelial injury, epigenetic variation, and inflammation) are more likely to be applied in clinical settings. Some studies have suggested and tested the combination of several biomarkers to explore the relationship with sepsis-related ARDS (97–101), with some success. For example, Zhao et al. (98) validated an ARDS-mortality prediction model, including the age, surfactant protein D, and interleukin-8, which may be useful for risk assessment in clinical trial enrollment. However, none of these candidate research schemes has yet been clinically applied in such patients. It is therefore important to further study and clarify the potential of these candidate schemes.

Our knowledge of sepsis-related ARDS disorders has grown over the last two decades, and our capacity to detect and treat such patients has steadily increased, saving the lives of a huge number of patients. The death rate of sepsis-related ARDS patients, on the other hand, remains at the forefront of many diseases, and long-term consequences for surviving patients are also a serious issue. Further research in the following areas could assist enhance patient outcomes to change this predicament. Exploring strategies to reduce lung endothelial and epithelial cell damage and finding ways to promote lung endothelial and epithelial cell repair is necessary from a molecular standpoint. On the clinical side, we will actively investigate particular biomarkers closely associated with the disease so that the disease may be diagnosed early in clinical work and early treatment intervention can be carried out to prevent the disease from progressing further.

## Author contributions

HG, YC, MC, and CL participated in the conception, drafting, and revision of the manuscript. All authors critically reviewed the paper. All authors have read and approved the final manuscript.

## Funding

This work was supported by grants from the Hainan Province Science and Technology Special Fund (ZDKJ202004 and ZDKJ2021038), National Natural Science Foundation of China (81871611), and Finance Science and Technology Program of Sichuan Province (2022YFS0602).

## Conflict of interest

The authors declare that the research was conducted in the absence of any commercial or financial relationships that could be construed as a potential conflict of interest.

## Publisher's note

All claims expressed in this article are solely those of the authors and do not necessarily represent those of their affiliated organizations, or those of the publisher, the editors and the reviewers. Any product that may be evaluated in this article, or claim that may be made by its manufacturer, is not guaranteed or endorsed by the publisher.
